# Supplementary material for: NOMe-HiC: joint profiling of genetic variant, DNA methylation, chromatin accessibility, and 3D genome in the same DNA molecule
Source: Genome Biol. 2023 Mar 16;24:50. doi: 10.1186/s13059-023-02889-x (PMC10018866; doi:10.1186/s13059-023-02889-x)
Supplement: Supplementary file 3 — Additional file 3. Review history. [file 13059_2023_2889_MOESM3_ESM.docx]

**Review history**

**First round of review**

**Reviewer 1**

In this manuscript, Fu et al, present a method to measure simultaneously the transcriptome, the long-range chromatin interactions, DNA methylation, as well as chromatin accessibility in cell populations. By resolving these modalities simultaneously, the authors ask if chromatin accessibility and TF binding on DNA are correlated with the formation of long-range interaction. Additionally, they use SNPs as a natural genetic perturbation of chromatin accessibility and long-range interaction. The development of such a method holds many promises as it can address long standing questions in the field, such as for instance how binding of TFs at regulatory elements contributes to the formation of long-range DNA interactions. While the manuscript shows proof of concept of the feasibility of such a joined measurement, it falls short in demonstrating that the data are usable to address these important biological questions. The current analysis only scratches the surface of the possibilities open by such an assay. It remains to be clarified whether this is due to technical limitations of the data (signal/noise in the accessibility measure; sequencing depth required…), or yet incomplete analysis of the data. Moreover, several QC controls and reproducibility analysis are missing. Below is a list of suggestions on how to improve the data analysis in order to prove the relevance of this new approach. In general, the manuscript is hard to read and will require significative improvements in wording of the text, further detailing figure legends to meet the standards of Genome Biology.


Major comments:
- There are many grammatical and spelling errors. Overall, language should be greatly improved to improve the readability of the manuscript. This should be revise throughout the manuscript (i.e. Line 72, 88…)
- Legends are often lacking critical details and should be revised throughout the manuscript.
- The authors show that TagMe-seq resolves long-range interactions similar to in-situ HiC at low resolution (>250kb). Yet supplementary figure 1d does not compare the methods at higher resolutions (<50kb or lower). This point needs to be addressed in order to grasp how resolutive is the TAGMe-seq compared to the reference HiC data.
- The reproducibility of the TAGMe-seq resolving the 3D structure of the chromatin is not clearly addressed (comparison replicates). The sequencing depth is very low for the replicate 1 in both cell lines (supplementary Table 2). Therefore, computing the reproductivity of the measured 3D genome interactions will require deeper sequencing of those samples.
- TAGMe-seq seems to overestimate the endogenous methylation (Fig1c). The authors should show that the GpC treatment does not interfere with the measurement of endogenous CpG methylation or provide an explanation as for why they observe such a bending in the signal. For instance, it is unclear if the CCG context that is methylated by McvIPI has been excluded from the analysis.
- The signal/noise ratio of the transcription factor footprints seems in general very low. For instance, the CTCF footprints presented in supplementary figure 2A are much lower than those detected in previous studies (Kelly et al, Sonmezer et al). Is that a consequence of the crosslink? If they want to claim that they can detect TF/Pol II footprints, the authors should demonstrate that the readout of protein occupancy is quantitative in these conditions (i.e. comparison to ChIP-seq). It is unclear why the authors decided to use z-score to look at TF footprint in of GC accessibility data (Figure1e). Methylation in GpC context should be displayed and they should demonstrate that those footprints quantitatively reflect protein occupancy. This also holds true for Pol II footprints that are not clearly visible on Figure 1d. It remains to be demonstrated if these are quantitively reflecting Pol II occupancy. Otherwise, these staements =should be removed and it should be clearly stated that the method is not quantitively resolving protein footprints.

- In Figure 2a the authors compare the co-accessibility at sites that are in contact in the genome. To that end they calculate the Pearson correlation of the average accessibility of individual DNA molecules that are in contact. Since the values appear to be largely binary (Figure 2a), Pearson correlation is not an appropriate way to test for co-accessibility as it only deals with continuous data. Moreover, the analysis is rather high level (comparing context, with random). It would be interesting to explore the distribution of co-accessibility at individual regions. Moreover, it would be critical to compare these measures over replicates and cell types. The current analysis is superficial. It is critical to demonstrate the ability of the method to robustly quantify co-accessibility at individual distant sites across the genome.

- -Figure 2, it would be important to illustrate the co-accessibility at individual loci and contrast it with regions where no co-accessibility is observed. Is co-accessibility happening for all regions in contact, is that a subset?
-
- Quality controls and descriptive statistics (methylation distribution at anchors, median of GC site in anchors, size of anchors, etc..), distribution of CG and GC methylation at both anchors would help the reader to make is opinion on the quality if the data.

- Additionally, the authors do not explore other features that they measure at the loops? For instance, if they can measure TF footprints, what is the co-binding frequency across distant sites (i.e. CTCF). What is the DNA methylation status at the looping sites… These analysis would demonstrate the usability of TAGMe-seq data to address biological questions.


- The authors focus the analysis on long range interactions (>20kb) which represent only a minor fraction of 3D chromatin interactions detected by TAGMe-seq (8,1% to 15.2% of the total contacts depending of the sample) (Figure 2b). What about the chromatin loops between 1kb-20kb? It would be good to clarify why these were excluded from the analysis.
- Why is the scale of the analysis different between Figure 2 and Figure 3. Here the analysis is focused on loops >1kb. This should be clarified or changed.
- In their current analysis, the authors associate presence of a SNP in a TFBS with loss of TF binding. Yet, the loss of TF binding should be demonstrated. This could be archived by analyzing the changes in TF footprints at SNP anchors of both alleles or analysis of ChIP-seq data in the same cell lines.
- It is great that the code developed to analyze the TAGMe-seq is provided in a GitHub repository. Yet, it is extremely difficult to relate it to the material and methods, preventing the reusability of the methodology. The code should be further commented, and a descriptive vignette should be provided to ensure reusability.

Minor comments:
- In supp 1b, c, e, the authors should explicitly state in the legend at which resolution the compartments, insulation scores and TADs have been computed.
- There is no description of the tools used to convert BAMs into .hic files (tools / parameters)
- supp table 2: The sum of the trans-contacts and cis-contacts for the sample IMR90, rep2 does not correspond to the total contacts number (393.110.844 when 400.278.562 is stated)
- The authors should precise the threshold used ('with GCH methylation levels' - line 303)
- p-value added to the scatter plots (p-value <2.2e-16) fig2a should be removed and is not directly linked with the data plotted
- The density of scatter should have a continues scale (Fig 2)
- The number of read pairs with computed GCH methylation levels in both ends should be added to supplementary table2.
- The number of pairs used to calculate the Pearson correlation in each group of loops (local and long-range) should be explicit on the figure or in the legend.
- 'Group 3 showed the highest depletion of TF binding sites that are usually bound to the promoter regions' (line 160). It is unclear to what data this refers to.
- : 'Since the sequencing reads come from the same single DNA molecules, the other ends of the read pairs were also separated into two alleles' (lines 145-147). It is unclear what the author mean her. Does this mean that only one of the read is assigned to a specific allele with a SNP and that the second one is assumed to be on the same allele (since most HiC pairs are in cis?)

**Reviewer 2**

This manuscript by Fu et al has developed a new method, TAGMe-seq, profiling of genetic variants, DNA methylation, chromatin footprints, 3D genome, and transcriptome. In general, this assay seems a very good approach as a single method, if this assay is validated in detail by comparing available data set by other approaches or assays on the same cell line (IMR-90), such as polymorphism, DNA methylation, gene expression, 3D genome, footprints (chromatin accessibility).
Here are some additional concerns regarding this assay:
1. The authors should show locus specific validation, such H19, as imprinting gene along with long noncoding RNA. In addition, authors should at least show one example locus of 3D genomic architecture near promoters or enhancers in detail, and how the transcripts and/or eRNAs are cross talked in this approach. I think these validations can help general readers to appreciate this assay.
2. Because of the obtained transcripts or RNA by cross-link, is the RNA level quantitative by comparing regular gene expression assay, such as RNA-seq? It should be validated. The authors fail to discuss this issue.
3. Furthermore, the available DNA sequence for this assay is after bisulfite treatment, it is very difficult to distinguish unmethylated C with T, the authors also fail to discuss this issue for their identification of genetic variants.
4. This method involves lots of treatment steps for the nuclei, are there any quality controls for each step? For example, for the GpC treatment, how do authors control for under- or over treatment? Since this is a new method developed, the author should provide more detailed description about how they performed quality control for each step of the method.

**Authors’ response to reviewers**

Comments from Reviewer 1

In this manuscript, Fu et al, present a method to measure simultaneously the transcriptome, the long- range chromatin interactions, DNA methylation, as well as chromatin accessibility in cell populations. By resolving these modalities simultaneously, the authors ask if chromatin accessibility and TF binding on DNA are correlated with the formation of long-range interaction. Additionally, they use SNPs as a natural genetic perturbation of chromatin accessibility and long-range interaction. The development of such a method holds many promises as it can address long standing questions in the field, such as for instance how binding of TFs at regulatory elements contributes to the formation of long-range DNA interactions. While the manuscript shows proof of concept of the feasibility of such a joined measurement, it falls short in demonstrating that the data are usable to address these important biological questions. The current analysis only scratches the surface of the possibilities open by such an assay. It remains to be clarified whether this is due to technical limitations of the data (signal/noise in the accessibility measure; sequencing depth required…), or yet incomplete analysis of the data. Moreover, several QC controls and reproducibility analysis are missing. Below is a list of suggestions on how to improve the data analysis in order to prove the relevance of this new approach. In general, the manuscript is hard to read and will require significative improvements in wording of the text, further detailing figure legends to meet the standards of Genome Biology.

Our overall response: Thanks for the comments and suggestions! Indeed, the current manuscript focuses on technology development rather than its application to biological questions. We only showed the proof of concept of the feasibility for future possible biological applications. Here, we added additional QC measurements, computational analysis, and more deeply sequenced biological replicates to demonstrate the reproducibility of the technology.

We also revised the whole manuscript to make it easier for readers to understand.

Major comments:

- There are many grammatical and spelling errors. Overall, language should be greatly improved to improve the readability of the manuscript. This should be revise throughout the manuscript (i.e.

Line 72, 88…)

- Legends are often lacking critical details and should be revised throughout the manuscript.

Our response: We apologize for these grammatical and spelling errors. We have revised the whole manuscript extensively. We change the assay name to NOMe-HiC since we think it might be better to reflect the origin of the assay.

- The authors show that TagMe-seq resolves long-range interactions similar to in-situ HiC at low resolution (>250kb). Yet supplementary figure 1d does not compare the methods at higher resolutions (<50kb or lower). This point needs to be addressed in order to grasp how resolutive is the TAGMe- seq compared to the reference HiC data.

Our response: Thanks for the suggestions. We sequenced another biological replicate in IMR- 90 and therefore increased the coverage of the library. We further compared our results with *in situ* Hi-C at the same cell types across different resolutions (250kb, 50kb, and 25kb). The results show consistent high similarity between our assay and *in situ* Hi-C across all of the resolutions (Figure 2a and Supplementary Figure 1d). For your convenience, we also provide it here:

Figure 2a

Supplementary Figure 1d

- The reproducibility of the TAGMe-seq resolving the 3D structure of the chromatin is not clearly addressed (comparison replicates). The sequencing depth is very low for the replicate 1 in both cell lines (supplementary Table 2). Therefore, computing the reproductivity of the measured 3D genome interactions will require deeper sequencing of those samples.

Our response: Thanks for the suggestions. Rep1 was generated in the very beginning during the technology development. Thus, we only sequenced them with shallow coverage. Now, we generated another deeply sequenced biological replicate in IMR-90 cell lines (rep3, ~900M unique mapped paired-end reads for DNA and ~400M unique mapped paired-end reads for RNA). The SCC from HiCRep is very high between the biological replicates across different resolutions (Supplementary Figure 8a). We add the analysis in reproducibility across all of the modalities in Supplementary Figure 8. For your convenience, we also provide it here.

- TAGMe-seq seems to overestimate the endogenous methylation (Fig1c). The authors should show that the GpC treatment does not interfere with the measurement of endogenous CpG methylation or provide an explanation as for why they observe such a bending in the signal. For instance, it is unclear if the CCG context that is methylated by McvIPI has been excluded from the analysis.

Our response: We apologize for the mistake here. The labels of the x-axis and y-axis were swapped by accident. We have corrected this in the new Figure 2b. The scatter plot now is similar to what we showed in the original Methyl-HiC paper. In Methyl-HiC, “more CpGs in open chromatin regions such as promoters and enhancers, likely due to reduced access to heterochromatin by the restriction enzyme” (see mESC result at Figure 1d in Li & Liu et al. 2019 Nature Methods, PMCID: PMC6765429). For your convenience, we also provide it here:

Figure 2b

Figure 1d at mESC in original Li & Liu et al. 2019 Nature Methods paper.

In the original manuscript, we only exclude GCG for the estimation of endogenous DNA methylation (HCG, H=A,C, or T). Since there is some residual methylation at CpC sites after M.CviPI treatment, in the revised manuscript, we also perform the comparison of DNA

methylation after excluding CCG (WCG, W=A or T). The concordance is still similar (see Supplementary Figure 1h). For your convenience, we also provide it here:

Supplementary Figure 1h

- The signal/noise ratio of the transcription factor footprints seems in general very low. For

instance, the CTCF footprints presented in supplementary figure 2A are much lower than those detected in previous studies (Kelly et al, Sonmezer et al). Is that a consequence of the crosslink? If they want to claim that they can detect TF/Pol II footprints, the authors should demonstrate that the readout of protein occupancy is quantitative in these conditions (i.e. comparison to ChIP-seq). It is unclear why the authors decided to use z-score to look at TF footprint in of GC accessibility data (Figure1e). Methylation in GpC context should be displayed and they should demonstrate that those footprints quantitatively reflect protein occupancy. This also holds true for Pol II footprints that are not clearly visible on Figure 1d. It remains to be demonstrated if these are quantitively reflecting Pol II occupancy. Otherwise, these staements should be removed and it should be clearly stated that the method is not quantitively resolving protein footprints.

Our response: Thanks for the comments. Indeed, due to the cross-linking step, the signal-to- noise ratio is lower than that in the original NOMe-seq/dSMF manuscript. To demonstrate the relationship to protein occupancy levels (Pol2 ChIP-seq), we also plot the ChIP-seq signals in the corresponding quantiles. (see Figure R2 and Supplementary Figure 2a). To demonstrate the relationship more directly, we plot the scatterplot between ChIP-seq signals and GpC accessibility level at PolII binding sites (mean value +/-250bp to the center of PolII ChIP-seq IDR peaks from ENCODE) (see Supplementary Figure 2b). For your convenience, we also provide it here.

Supplementary Figure 2b

The reason we use the z-score is that the global GpC accessibility level is confounded by multiple factors, such as GpC methyltransferase treatment concentration, treatment time, nuclei permeability for the enzyme, native chromatin environment, and other random noise. To better compare the footprint levels at the same regions across different cell types, the z-score is likely better to show, since the global background will be normalized almost to 0. We also attach the plot without z-score transformation here. The results still show similar patterns here but with shifted values. For your convenience, we also show Fig R3 here:

- In Figure 2a the authors compare the co-accessibility at sites that are in contact in the genome. To that end they calculate the Pearson correlation of the average accessibility of individual DNA molecules that are in contact. Since the values appear to be largely binary (Figure 2a), Pearson correlation is not an appropriate way to test for co-accessibility as it only deals with continuous data. **Our response: Thanks for the suggestions. To demonstrate the correlation/association between two binary vectors, we add additional metrics: phi coefficient (also called Matthews correlation coefficient (MCC)) and Tetrachoric Correlation, both of which are specifically designed to measure the correlation between two binary vectors. Since methylation in some regions are still continuous, we binarize them by a naive cut-off (<0.5 will be 0 and >=0.5 will be 1). All the new results still suggest a high concordance of accessibility at the loop contact regions (see new Figure 3b). For your convenience, we also show it here:**

Figure 3b

Moreover, the analysis is rather high level (comparing context, with random). It would be interesting to explore the distribution of co-accessibility at individual regions.

Our response: Thanks for the suggestions. We have to admit that there are some limitations of the current genome-wide approach to understanding the concordance between specific pairs of CREs. (1). Even after merging with a newly sequenced library, the resolution in the merged library is still low (~20kb resolution) to understand the dynamics at the scale of individual paired CREs (10-100bp scale). (2). This is an assay on bulk cells, and only regions (distant to each other) in spatial proximity are profiled. And we know that the interactions between CREs are dynamic across the cell populations. That is: between two distant CREs, for example, CEBPB and Pol2, only CEBPB and Pol2 that is spatially close in the sub-group of cells are mostly profiled due to the cellular heterogeneity. For the same CEBPB-Pol2 distant paired region that does not interact in the other sub-group of cells, we cannot get read pairs between them. Long-read sequencing that covered the two regions (sampling from all cells) combined with our NOMe-HiC technology (sampling mostly from cells that have interactions between two distant regions) with enough read pairs at these two regions can provide an overview of how often these two CREs are concordant with each other. The situation here is different from dSMF. dSMF utilizes the paired-end read sampling from all of the cells in the specific local regions, which can easily give the estimation of the relative concordance level between two CREs, which, however, have to be linearly close to each other due to the short reads. Our technology here provided a proof-of-concept answer to the question: spatial proximal regions are concordant with each other in epigenetics (both DNA methylation and GpC methyltransferase footprint).

Nonetheless, to address this concern, we have added a locus plot at individual regions to show some proof-of-concept examples (see Figure 3c):

Figure 3c

We also performed analysis between chromHMM states regions by integrating the concordance level and interaction frequency (by summing up the absolute differences of GCH methylation between the two ends). We can clearly see that the chromHMM states are separated into three different groups (see Figure below). We repeated the same analysis between pairs of TFBS and observed some patterns between paired CREs groups (for

example, Pol2 and its often distantly interacting TFs, CTCF always together with the cohesins RAD21 and SMC3). However, we believe that these analyses on the current technology are still confounded by the interaction frequency between two paired regions and therefore we did not include them in the current manuscript. Future targeted deep NOMe-HiC combined with long- read sequencing will finally answer this question more confidently.

Moreover, it would be critical to compare these measures over replicates and cell types. The current analysis is superficial. It is critical to demonstrate the ability of the method to robustly quantify co- accessibility at individual distant sites across the genome.

We have added additional analysis to demonstrate the reproducibility over replicates (see Supplementary Figure 9) and across cell types (Figure 3b and Supplementary Figure 9). We also show them here for your convenience. The GCH concordance is consistently high across replicates and cell types compared to shuffled reads in the same regions. However, we do observe some variation between cell types, which suggests potential cell type specificity.

Figure 3b

Supplementary Figure 9

- -Figure 2, it would be important to illustrate the co-accessibility at individual loci and contrast it with regions where no co-accessibility is observed. Is co-accessibility happening for all regions in contact, is that a subset?

Our response: Thanks for the comment. As we explained in the previous answer, the current technology is limited for answering this question at individual loci. Similar to local co- accessibility, the results suggest that long-range co-accessibility is a subsect of all regions that are in contact. Otherwise, the long-range correlation we obtained here will always be almost 1 across cell types and replicates.

- Quality controls and descriptive statistics (methylation distribution at anchors, median of GC site in anchors, size of anchors, etc..), distribution of CG and GC methylation at both anchors would help the reader to make is opinion on the quality if the data.

Our response: Thanks for the suggestions. When we call the loops, we utilize the fixed threshold of 25kb as the parameter to call chromatin loops. Therefore, the sizes of the anchors

are all 25kb. We added other additional QC and descriptive analysis to Supplementary Figure

11. For your convenience, we also show it here:

We add several additional QC steps in the experimental and computational side (details in Method).

To control for under- or over-treatment, we examined GCH accessibility at several negative and positive control regions (Supplementary Fig. 4). We followed the same treatment time and enzyme concentration as that in the original NOMe-seq paper - the accessibility at control regions was largely the same as before. To make sure the nuclei are still in good shape, we examined nuclei status after each step (see Supplementary Figure 7): cross-linking, restriction enzyme digestion, biotin fill-in, ligation, and GpC methyltransferase treatment. After bisulfite conversion, we measured DNA quantity and quality with BioAnalyzer 2000 before and after library construction. Before deep sequencing, we sequenced the library to shallow coverage using the Illumina MiSeq platform to check the quality of the DNA and RNA libraries.

In computational analyses, we examined the QC metrics for each molecule measurement, as was done in the previous studies: (1). For DNA methylation, we examined the bisulfite conversion rate. (2). For GCH accessibility, we examined GCH methyltransferase efficiency and endogenous DNA methylation levels at important regulatory elements, such as CGI promoters and CTCF binding sites. We also examined the average GCH accessibility globally in autosome and mitochondria DNA. (3). For 3D genome measurements, we examined the fraction of cis- long-range interactions and trans- interactions. (4). For genetic variant calling, we examined the Ti/Tv ratio. (5). For RNA, we utilized RNASeQC226 to summarize the RNA QC.

(6). We also examined the unique mapping efficiency, PCR duplication rate, and library complexity, as all other common NGS data metrics using PICARD, FastQC, and MultiQC27 tools

However, for the CpG and GpC methylation distribution in anchors, Since the anchor size is 25kb, the average methylation in anchors mostly reflects the global DNA methylation level (see figure below), even by using public WGBS at IMR90 from ENCODE (grey, blue and green

color). We are not completely sure the purpose of using CG and GC methylation distribution in anchors for the QC.

- Additionally, the authors do not explore other features that they measure at the loops? For instance, if they can measure TF footprints, what is the co-binding frequency across distant sites (i.e. CTCF). What is the DNA methylation status at the looping sites… These analysis would demonstrate the usability of TAGMe-seq data to address biological questions.

Our response: Thanks for the suggestions. For the analysis of co-binding frequency at CREs or TF, we have already described the limitation of our technology in the previous answer.

For the analysis of DNA methylation status at looping sites, our technology is based on Methyl-HiC. We performed a comprehensive analysis of the methylation concordance at looping sites in the previous manuscript (Li & Liu et al. Nature Methods 2019). Therefore, we did not repeat the analysis here. For your convenience, we also attach a similar analysis on DNA methylation by using WCG here (also see Supplementary Figure 10):

- The authors focus the analysis on long range interactions (>20kb) which represent only a minor fraction of 3D chromatin interactions detected by TAGMe-seq (8,1% to 15.2% of the total contacts depending of the sample) (Figure 2b). What about the chromatin loops between 1kb-20kb? It would be good to clarify why these were excluded from the analysis.

Our response: Thanks for the comment. The distance cut-off is indeed arbitrary. We follow the most frequently used cut-off in 3D genome analysis for long-range cis-interactions (e.g. PMID: 28135255). In traditional Hi-C analysis, people usually exclude the interactions between 1kb- 20kb to avoid the potential artifacts of self-ligation. Therefore, in this proof-of-concept technology study, we exclude the same regions as others did for 3D genome analysis.

- Why is the scale of the analysis different between Figure 2 and Figure 3. Here the analysis is focused on loops >1kb. This should be clarified or changed.

Our response: We apologize for the mistake that made the reviewer confused here. In the revised manuscript, we make this consistent between Figure 2 and 3 (new Figure 3 and 4) (only using interactions >20kb).

- In their current analysis, the authors associate presence of a SNP in a TFBS with loss of TF binding. Yet, the loss of TF binding should be demonstrated. This could be archived by analyzing the changes in TF footprints at SNP anchors of both alleles or analysis of ChIP-seq data in the same cell lines.

Our response: Thanks for the suggestions. We have added additional extensive allelic- dependent TF binding analysis (using allelic imbalance of ChIP-seq reads performed in GM12878) at these loci to validate the loss of TF binding there. Indeed, we observe strong enrichment for allele-dependent TF binding at the loci identified by NOMe-HiC (see Supplementary Figure 13). For your convenience, we also show it here:

- It is great that the code developed to analyze the TAGMe-seq is provided in a GitHub repository. Yet, it is extremely difficult to relate it to the material and methods, preventing the reusability of the methodology. The code should be further commented, and a descriptive vignette should be provided to ensure reusability.

Our response: We apologize for the incomplete guides at the GitHub repository. We updated the tutorial method there for others to use it more conveniently.

Minor comments:

- In supp 1b, c, e, the authors should explicitly state in the legend at which resolution the compartments, insulation scores and TADs have been computed.

Our response: Thanks for the suggestion. Originally, we described it in the Method section (Analysis of in situ Hi-C data). Here, we also added the resolution in the legend of Supp 1b,c,e,f.

- There is no description of the tools used to convert BAMs into .hic files (tools / parameters)

Our response: We apologize for this. The conversion of bam files to .hic files was done by a customized python script (sam2juicer.py) from our previous Methyl-HiC paper (Li & Liu et al. 2019 Nature Methods). The link to the script is here: https://bitbucket.org/dnaase/bisulfitehic/src/master/src/python/sam2juicer.py

We also add the description in the Methods section of the manuscript.

- supp table 2: The sum of the trans-contacts and cis-contacts for the sample IMR90, rep2 does not correspond to the total contacts number (393.110.844 when 400.278.562 is stated)

Our response: We apologize for the mistake here. We corrected the number in Supplementary Table 2.

- The authors should precise the threshold used ('with GCH methylation levels' - line 303)

Our response: We are sorry about the mistake here. We meant read pairs with at least three

GCH sites on both ends. We have corrected this in the revised manuscript.

- p-value added to the scatter plots (p-value <2.2e-16) fig2a should be removed and is not directly linked with the data plotted

Our response: Thanks for the suggestions. We removed the p-value at Figure 2a (new Figure 3a).

- The density of scatter should have a continues scale (Fig 2)

Our response: Thanks for the suggestion. The density key only represented the relative density within the heatmap. The absolute value about the density is not important for the figure and may be misleading to the reader (they may try to compare the density between different heatmaps in Fig 2a, the new Fig 3a, which is meaningless). Therefore, we only marked the direction (low->high) in the density key.

- The number of read pairs with computed GCH methylation levels in both ends should be added to supplementary table2.

Our response: Thanks for the suggestion. We added the corresponding statistics in Supplementary Table 2.

- The number of pairs used to calculate the Pearson correlation in each group of loops (local and long-range) should be explicit on the figure or in the legend.

Our response: Thanks for the suggestion. We added the corresponding statistics to the corresponding figure legend (Figure 3b and Supplementary Figure 9). For the short-range mode, to reduce the time cost (many more local pairs), we randomly sampled the read pairs (up to 100 pairs in each local region). That is why the number of pairs in short-range is similar between merged, rep2, and rep3.

- Group 3 showed the highest depletion of TF binding sites that are usually bound to the promoter regions' (line 160). It is unclear to what data this refers to.

Our response: We are sorry about the imprecise description. We revised the sentence in the manuscript. For your convenience, we also attach it here:

“Group 1 showed the highest enrichment for CTCF binding sites at both anchors (Fig. 4b, upper panel), suggesting that disruption of the CTCF binding by genetic variants at the SNP anchor may also affect the loop structure, and thus, the binding activities at the non-SNP anchor in spatial proximity. Group 2 exhibited the highest enrichment of CTCF and PolII binding sites at the SNP anchors, but enrichment of different TFs at the non-SNP anchors, which usually bind to distal enhancer regions (Fig. 4b, middle panel). Group 3 showed the highest depletion of TF binding sites that are usually bound to the promoter regions (Fig. 4b, bottom panel).”

- Since the sequencing reads come from the same single DNA molecules, the other ends of the read pairs were also separated into two alleles' (lines 145-147). It is unclear what the author mean her. Does this mean that only one of the read is assigned to a specific allele with a SNP and that the second one is assumed to be on the same allele (since most HiC pairs are in cis?)

Our response: We are sorry about the imprecise description here. Yes, this is what we meant. We also revised the sentence in the manuscript to make it more clear. For your convenience, we also attach it here:

“We separated the distantly connected NOMe-HiC read pairs into two groups by their parent- of-origin at the heterozygous SNPs overlapped in one end (SNP anchors, Fig. 4a). Since the NOMe-HiC read pairs mostly come from the same DNA molecules, the other ends of the read pairs were also separated into two alleles (although they do not necessarily overlap with heterozygous SNPs) and mapped over large genomic distances to the SNP anchors (non-SNP anchors, Fig. 4a).”

Comments from Reviewer 2

This manuscript by Fu et al has developed a new method, TAGMe-seq, profiling of genetic variants, DNA methylation, chromatin footprints, 3D genome, and transcriptome. In general, this assay seems a very good approach as a single method, if this assay is validated in detail by comparing available data set by other approaches or assays on the same cell line (IMR-90), such as polymorphism, DNA methylation, gene expression, 3D genome, footprints (chromatin accessibility).

Here are some additional concerns regarding this assay:

1. The authors should show locus specific validation, such H19, as imprinting gene along with long noncoding RNA. In addition, authors should at least show one example locus of 3D genomic architecture near promoters or enhancers in detail, and how the transcripts and/or eRNAs are cross talked in this approach. I think these validations can help general readers to appreciate this assay.

Our response: Thanks for the suggestions. We add locus specific example together with public dataset across different modalities at a genome browser view (see new Supplementary Figure 6 and Figure 3c). For your convenience, we also show it here:

And co-accessibility at imprinting regions:

Figure 3c

2. Because of the obtained transcripts or RNA by cross-link, is the RNA level quantitative by comparing regular gene expression assay, such as RNA-seq? It should be validated. The authors fail to discuss this issue.

Our response: Thanks for the comments. In the original manuscript, we have already compared the RNA with the total RNA-seq data from the same cell lines (see Fig 2e and

Supplementary Figure 1j). Since we performed transcriptome analysis at two different cell types, we also have the comparison about the differential expressed genes detected by NOMe- HiC and traditional total RNA-seq (see Supplementary Figure 5).

3. Furthermore, the available DNA sequence for this assay is after bisulfite treatment, it is very difficult to distinguish unmethylated C with T, the authors also fail to discuss this issue for their identification of genetic variants.

Our response: Thanks for the comments, and sorry about the lack of description in the original manuscript. We developed Bis-SNP before to identify the genetic variants in bisulfite- treated DNA sequences (Liu et al. 2012 Genome Biology). However, the accuracy is still not enough for the genome-wide SNP calling. In principle, we utilize the same strategy as Bis- SNP. We improve the original Bis-SNP approach by filtering out loci with a high error rates.

Further, we pre-phase and impute the genotypes by using an external reference panels of SNPs in the population, such as the 1000 genome project. The results suggest similar accuracy now as that in WGS. We added these descriptions to the method section of the revised manuscript.

4. This method involves lots of treatment steps for the nuclei, are there any quality controls for each step? For example, for the GpC treatment, how do authors control for under- or over treatment? Since this is a new method developed, the author should provide more detailed description about how they performed quality control for each step of the method.

Our response: Thanks for the suggestions. We indeed performed QC at each step. To control for the under- or over-treatment, we will check the GpC accessibility at several negative and positive control regions. Similar to the original NOMe-seq paper, we will check the accessibility level at negative control and positive control regions (see Supplementary Figure 4). If it is over-treated, the negative control region will possibly be accessible. If it is under treatment, the positive control regions will show a few accessibilities there. We follow the same treatment time and enzyme concentration as that in the original NOMe-seq paper, the accessibility at control regions is largely similar to before. For your convenience, we also show it here:

To make sure the nuclei are still in good shape, we always examined the nuclei status after each step (see Supplementary Figure 7, for your convenience, it is attached here): cross- linking, restriction enzyme digestion, biotin fill-in, ligation, GpC methyltransferase treatment.

After bisulfite conversion, we measured DNA quantity and quality with BioAnalyzer 2000 before and after library construction. Before deep sequencing, we sequenced the library to shallow coverage using the Illumina MiSeq platform to check the quality of the DNA and RNA libraries.

In computational analyses, we examined the QC metrics for each molecule measurement, as was done in the previous studies: (1). For DNA methylation, we examined the bisulfite conversion rate at non-CpG site in autosomes, mitochondria DNA, and spike-in lambda DNA.

(2). For GCH accessibility, we examined GCH methyltransferase efficiency and endogenous DNA methylation levels at important regulatory elements, such as CGI promoters and CTCF binding sites. We also examined the average GCH accessibility globally in autosome and

mitochondria DNA. (3). For 3D genome measurements, we examined the fraction of cis- long- range interactions and trans- interactions. (4). For genetic variant calling, we examined the Ti/Tv ratio. (5). For RNA, we utilized RNASeQC2 to summarize the RNA QC. (6). We also examined the unique mapping efficiency, PCR duplication rate, and library complexity, as all other common NGS data metrics using PICARD, FastQC, and MultiQC tools.

**Second round of review**

**Reviewer 1**

The authors have considerably revised their manuscript. They improved the quality of their data, displayed required quality controls and clarified the possibilities and limitations of their method.
Minor comments:
• Supplementary Figure 2: Figure name is misleading as Pol II and not TFs are displayed.
• I would suggest to add a section that discuss the current limitations of the methods in order to not create the wrong expectations to the readership. For instance the challenges linked to the quantification of co-occupancy/co-accessibility, the exploration of single sites or the quantification of TF/Pol II footprints should be clearly discussed in the main text. Some of these are discussed in the rebuttal and could be useful for the reader:
We have to admit that there are some limitations of the current genome-wide approach to understanding the concordance between specific pairs of CREs. (1). Even after merging with a newly sequenced library, the resolution in the merged library is still low (~20kb resolution) to understand the dynamics at the scale of individual paired CREs (10-100bp scale). (2). This is an assay on bulk cells, and only regions (distant to each other) in spatial proximity are profiled. And we know that the interactions between CREs are dynamic across the cell populations. That is: between two distant CREs, for example, CEBPB and Pol2, only CEBPB and Pol2 that is spatially close in the sub-group of cells are mostly profiled due to the cellular heterogeneity. For the same CEBPB-Pol2 distant paired region that does not interact in the other sub-group of cells, we cannot get read pairs between them. Long-read sequencing that covered the two regions (sampling from all cells) combined with our NOMe-HiC technology (sampling mostly from cells that have interactions between two distant regions) with enough read pairs at these two regions can provide an overview of how often these two CREs are concordant with each other. The situation here is different from dSMF. dSMF utilizes the paired-end read sampling from all of the cells in the specific local regions, which can easily give the estimation of the relative concordance level between two CREs, which, however, have to be linearly close to each other due to the short reads. Our technology here provided a proof-of-concept answer to the question: spatial proximal regions are concordant with each other in epigenetics (both DNA methylation and GpC methyltransferase footprint).

**Reviewer 2**

The authors have answered most my concerns.

**Authors’ response to reviewers**

Referee #1:

1. We have incorporated the concerns from Referee 1 into the Discussion part.

2. We have updated the Supplementary Figure 2's Figure name.
